# Chromosome Replacement and Deletion Lead to Clonal Polymorphism of Berry Color in Grapevine

**DOI:** 10.1371/journal.pgen.1005081

**Published:** 2015-04-02

**Authors:** Frédérique Pelsy, Vincent Dumas, Lucie Bévilacqua, Stéphanie Hocquigny, Didier Merdinoglu

**Affiliations:** 1 INRA, UMR1131, Colmar, France; 2 Université de Strasbourg, UMR1131, Strasbourg, France; MicroTrek Incorporated, United States of America

## Abstract

Clonal polymorphism mainly results from somatic mutations that occur naturally during plant growth. In grapevine, arrays of clones have been selected within varieties as a valuable source of diversity, among them clones showing berry color polymorphism. To identify mutations responsible for this color polymorphism, we studied a collection of 33 clones of Pinot noir, Pinot gris, and Pinot blanc. Haplotypes of the L2 cell layer of nine clones were resolved by genotyping self-progenies with molecular markers along a 10.07 Mb region of chromosome 2, including the color locus. We demonstrated that at least six haplotypes could account for the loss of anthocyanin biosynthesis. Four of them resulted from the replacement of sections of the ‘colored’ haplotype, sized from 31 kb to 4.4 Mb, by the homologous sections of the ‘white’ haplotype mutated at the color locus. This transfer of information between the two homologous sequences resulted in the partial homozygosity of chromosome 2, associated in one case with a large deletion of 108 kb-long. Moreover, we showed that, in most cases, somatic mutations do not affect the whole plant; instead, they affect only one cell layer, leading to periclinal chimeras associating two genotypes. Analysis of bud sports of Pinot gris support the hypothesis that cell layer rearrangements in the chimera lead to the homogenization of the genotype in the whole plant. Our findings shed new light on the way molecular and cellular mechanisms shape the grapevine genotypes during vegetative propagation, and enable us to propose a scheme of evolutionary mechanism of the Pinot clones.

## Author Summary

Pinot is one of the most ancient grapevine varieties made up of a large panel of clones, most of them used to produce very different wines with specific oenological characteristics in different vineyards around the world. This great diversity of clones, which is due to spontaneous somatic mutations that have occurred over time, makes Pinot a fascinating subject of study. It is the reason why we have undertaken a study focused on the color locus to identify the mutations responsible for color variation in a large panel of Pinot gris and Pinot blanc clones. The results we obtained shed light on large-scale molecular events that account for the loss of anthocyanin biosynthesis, such as chromosome replacement and deletion. These mutations first multiplied and, depending on the cell layer in which they occurred, lead to chimeras. Occasionally, cell layer rearrangements homogenize the whole plant. Clonal polymorphism of grapevine varieties results from a succession of such molecular and cellular mechanisms that are the driving forces behind the genetic drift of clones and the evolution of the grapevine genome.

## Introduction

Grapevine varieties display a wide palette of berry color, which ranges from the less pigmented green-yellow to the highest pigmented blue-black at maturity. At the molecular level, berry color is probably the best-documented trait in grapevine. The color phenotype of grape is due to the expression of *VvmybA1* and *VvmybA2*, two genes encoding transcription factors that regulate the anthocyanin pathway [1–3]. *VvmybA1* and *VvmybA2* are located in a single cluster of four *Myb* and *Myb*-like genes [4], spanning a 200 kb-region located on chromosome 2 [5]. The lack of anthocyanin pigments has been associated with the insertion of *Gret1*, a 10,422 bp retrotransposon, in the promoter region of *VvmybA1* combined with two mutations in the coding sequence of *VvmybA2*, a point mutation and a 2-bp deletion (CA) altering the reading frame. Mutations in both genes lead to the loss of transcription factor expression and consequently prevent anthocyanin biosynthesis [6]. Kobayashi et al. have shown that 10 colored varieties, including Pinot noir, were heterozygous at the color locus associating the ‘colored’ haplotype with the functional *VvmybA* genes and the ‘white’ haplotype. Conversely, they showed that 12 white varieties, including Pinot blanc, were homozygous for the ‘white’ haplotype [1]. Later, Fournier-Level et al. confirmed the prevalence of the ‘white haplotype’ in 141 *V*. *vinifera* varieties chosen to maximize the agro-morphological diversity of the cultivated compartment by identifying only nine varieties homozygous for the ‘colored’ haplotype, all corresponding to highly colored wine varieties [4]. The insertion of *Gret1* upstream from *VVMybA1* gene is considered as a ‘recent’ event [7] and may have occurred once in *V*. *vinifera* during grape domestication some 7,000 years ago. Later, this insertion spread among varieties by crossings in the cultivated compartment, possibly playing a key role in grape cultivation [8]. This hypothesis is supported by the fact that this insertion has not been detected in any of the North American or East Asian *Vitis* species [9].

Vegetative propagation is a conservative strategy used to obtain clones that are genetically identical copies of an original seedling. However, somatic mutations may occur naturally in the regenerative cells that give rise to the clones leading to clonal polymorphism [10]. In most cases, somatic mutations do not affect the whole plant; instead, they affect only one cell layer, leading to periclinal chimeras. Such structures are specific types of genetic mosaic in which one or two entire cell layers of the apical meristem are genetically distinct from the others and remain developmentally independent from the adjacent layers [11]. Periclinal chimeras do not threaten the plant’s fitness and are stable through vegetative propagation. Occasionally, cellular rearrangements in the chimera lead to homogenization of the genotype of the whole plant. Since its domestication, human beings have propagated grapevine vegetatively, for the oldest varieties, across multiple plant generations. As a result of these molecular and cellular mechanisms, divergent genotypes and, to some extent, divergent phenotypes may appear and this array of clones are a valuable source of diversity [12].

The grapevine genome sequence [13,14] is an invaluable resource to characterize the molecular nature of somatic mutations. New generation sequencing was used to compare a large portion of the genome of three Pinot noir clones selected for their phenotypic differences [15]. These authors identified three types of polymorphism (SNPs, Indels, mobile elements) and concluded that insertion polymorphism generated by mobile elements constitutes the most frequent mutational events with respect to clonal variation.

Clonal polymorphism affecting berry color has been intensively investigated in different varieties. It is observed in colored varieties such as Aramon, Grenache, Pinot or Terret, for which certified clones are available for blue-black, grey and green-yellow-skinned varieties [16] as well as in Cabernet Sauvignon [17]. Conversely, green-yellow-skinned berried varieties such as Savagnin, Chardonnay or Chasselas, can comprise pigmented clones. Clonal difference between a blue-black Pinot noir and a green-yellow Pinot blanc was shown to be caused by a large deletion, over 260 kb-long, that removed both functional *VvmybA* genes of the colored allele, leading to the green-yellow phenotype of Pinot blanc [17,18]. Similarly, a large deletion in the colored allele of Cabernet Sauvignon is responsible for the green-yellow phenotype of Shalistin berries, a bud sport of Cabernet Sauvignon [17]. Considering the grey-skinned phenotype of Pinot gris and Malian, a bud sport of Cabernet Sauvignon, it is now well established that it results from a chimeric structure composed of a colored L1 epidermis and L2 cells with a mutation in the color locus that prevents anthocyanin synthesis [17,19]. As the replacement of one cell layer by another can lead to the homogenization of the genotype of the whole plant, a two-step process has been proposed to explain the appearance of Pinot blanc and Shalistin. In the first step, a somatic mutation at the color locus impairing the anthocyanin biosynthesis could have affected one cell of the shoot apical meristem. Next, the mutation may be propagated by cell division to the entire L2 cell layer, creating the stable chimeras Pinot gris and Malian. In the second step, the invasion of epidermal colored cells (L1) by subepidermal white cells (L2) mutated at the berry-color locus could have led to the homogenization of the genotype of the white clones [17,19]. By investigating the structural dynamics along chromosome 2 of a set of 29 Pinot clones, Vezzulli et al. (2012) concluded that mutations impairing the color locus were deletions, ranging from 100 kb to 179 kb for two Pinot blanc clones, while ranging from 4,202 kb to 4,350 kb in the L2 cell layer of two Pinot gris clones leading to hemizygosity. This result led them to propose that Pinot blanc is not a bud sport of Pinot gris. They have subsequently proposed a novel parallel evolutionary model where the blue-black-skinned ancestor Pinot noir gave rise to the grey-skinned and the white-skinned berry mutants independently [20].

Pinot is thought to be one of the most ancient variety groups. Pinot shows primitive morphological characteristics analogous to those of the wild type ssp. *silvestris*, and is thus considered as “archaic” [21]. The Roman agricultural writer Columella cited a variety, present in Burgundy at the time of the Roman conquest, which may be Pinot [22]. Nowadays, wines produced from Pinot noir are among the most famous in the world. The age of the genotype, the total acreage planted in different vineyards around the world to produce very different wines with specific oenological characteristics and a possible proclivity toward spontaneous mutation [23] can explain the wide range of clones currently available within the Pinot group, among them Pinot gris and Pinot blanc. Consequently, the Pinot group is a good candidate for studying grapevine intravarietal diversity.

Here, we investigate a collection of Pinot noir, Pinot gris, and Pinot blanc clones, including two sets of Pinot bud sports, to identify molecular mechanisms leading to clonal polymorphism at the color locus. By resolving the structure of both haplotypes of nine clones along a region of the terminal arm of chromosome 2 extending over 10 Mb, we demonstrated that extended chromosomal structural changes, which could result from gene conversion are responsible for the color impairing of Pinot gris and Pinot blanc clones. We finally proposed a model integrating both mutations and cell layer rearrangements to explain the mechanism of clone diversification.

## Materials and Methods

### Plant material

The plant material consisted of a collection of 33 Pinot accessions phenotyped for berry color according to the OIV descriptor 225 (1: green-yellow; 4: grey; 6: blue-black) [24], among them clones certified by the French government authorities through a period of sanitary and genetic selection preserved in the French national repository (ENTAV, Le Grau du Roi, France). It consisted of five Pinot noirs (OIV 225: 6), among them three certified clones (PN162, PN292, PN871), five Pinot gris (OIV 225: 4), among them 2 certified clones (PG52, PG53), and 18 Pinot blanc (OIV 225: 1), among them 2 certified clones (PB54, PB55). In addition, two Pinot gris (BCPG9.S7.1 and PGMA19) and their respective bud sports, either green-yellow (BCPG9.S7.2 and PGMA19.S5) or blue-black-skinned (PGMA19.S6) were added. The non-certified Pinot clones were recovered from field selections in Alsace and Burgundy and preserved in germplasm repositories at the Institut National de la Recherche Agronomique (Colmar, France) and ATVB Mont Batois (Burgundy, France) (S1 Table).

Self-progenies were produced by self-fertilization of one Pinot noir (PN162), 3 Pinot gris (PG52, PG53 and PG3106), and five Pinot blanc (PB54, PB55, PB3009, PB3172, PB3232) clones.

The self-progenies of PN162, PG52 and PG53 were grown in the greenhouse as described in Blasi et al. [25] and brought to fructification. Fertile individuals were phenotyped for berry color according to the OIV descriptor 225.

Embryogenic cultures of clone PG52 and PG53 were initiated from the upper part of floral buds and plantlets regenerated from somatic embryos were grown according to Hocquigny et al. (2004) [19]. Brought to fructification in greenhouse, fertile plants were phenotyped for berry color according to the OIV descriptor 225.

### DNA extraction

Young expanded leaves of shoot tips of all individual field-grown (80–100 mg) were ground into fine powder with liquid nitrogen. Total DNA was extracted with the Qiagen DNeasy Plant mini-kit (Qiagen, Hilden, Germany), as described by the supplier. DNA from pith wood was extracted with the same procedure.

### Molecular analysis

Two sets of nuclear SSR markers were used. One set of 51 markers scattered on all of 19 linkage groups of the grapevine genome [26] was used to verify that clones in study were true-to-type Pinot (S2 Table). Another set of 13 markers grouped on the terminal extremity of chromosome 2 was used to investigate polymorphism in this region, among them VVNTm loci [4] (S3 Table). In addition SSR markers P2-106, P2-298 and P2-442 were mined from the grapevine sequence between loci SC8_0146_010 and VVNTm1 and primers were designed using Primer3 software [27] (S4 Table). All SSR markers were amplified using one 6-FAM, HEX or NED fluorophore-labelled primer (PE Applied Biosystems, Warrington, UK). PCR amplifications were carried out according to Hocquigny et al. (2004). PCR fragments were resolved on an automated 310C ABI PRISM DNA sequencer (PE Applied Biosystems, Foster City, CA), and sized with an ROX labeled-internal standard (50–654 bp) (PE Applied Biosystems, Foster City, CA). SSR alleles were scored using GenScan (version 3.1) and Genotyper (version 2.5.2) software (PE Applied Biosystems, Foster City, CA). They were named according to their size in base pairs.

Full and empty sites of the retrotransposon *Gret1* insertion site were amplified according to Kobayashi et al. (2004). *VvMybA2* primers were designed to specifically amplify the 2-bp deletion (CA) of the white allele [6]. Deletion was confirmed by sequencing. *Noble225*, a full-length copy of the Noble retrotransposon family [7] was mined by a Blat research [28] between loci VVNTm5 and VVIu20.1 on chromosome 2 and primers were designed to amplify the full and empty sites of the *Noble225* insertion. All primers were designed using Primer3 software [27] (S4 Table).

### Genetic mapping

Fifty-one polymorphic SSR markers from marker sets VVS [29], VVMD [30,31], VrZAG [32], VMC (Vitis Microsatellite Consortium, Agrogene, Moissy Cramayel, France), VVI [33] were used to analyze the entire S1 PN162 mapping population comprising 97 individuals. For mapping purposes, the same segregation pattern was assigned to all markers (<hkxhk>: locus heterozygous in both parents, two alleles), and genotypes were encoded (hh,hk,kk) for co-dominant loci and (k-,hh) for dominant loci, following JoinMap 3.0 data entry notation [34]. The berry color was encoded as a dominant locus, hh for OIV 225:1 (green-yellow) and k- for OIV 225: 6 (blue-black) and the *Gret1* insertion as a co-dominant marker.

Linkage analysis was performed as described in Blasi et al. [25] with JoinMap 3.0 [34].

## Results

### All accessions are true-to-type Pinot clones

In order to confirm that the 33 accessions selected were true-to-type Pinot clones, they were genotyped at 51 microsatellites loci scattered on the 19 linkage groups of the grapevine genome [26], except those mapped on the terminal part of chromosome 2. Twenty-two clones shared the same genotype, among them five blue-black-skinned, three grey-skinned, and 14 green-yellow-skinned accessions (S2 Table). The 11 remaining accessions displayed seven variant genotypes that differed from the typical genotype by the addition of one allele at one locus for six genotypes and at two loci for one genotype leading in most cases to tri-allele profiles characterizing a chimeric state (S5 Table). These results confirmed that all selected accessions were true-to-type Pinot clones.

### Detailed analysis of polymorphism along chromosome 2

Polymorphism in the distal region of chromosome 2, which includes the color locus, was investigated using 13 microsatellite loci mapped on a 10.07 Mb-long physical region, between loci VMC5g7 (position: 8.20 Mb) and VMC7g3 (position: 18.27 Mb) of chromosome 2. The total length of chromosome 2 is estimated to 18.78 Mb-long according to the 12X genome sequence (http://www.genoscope.cns.fr/externe/GenomeBrowser/Vitis). In addition to microsatellite marker polymorphism, insertion polymorphism of the retrotransposon *Gret1* present at the color locus in position 14.24–14.25 Mb [1] was used as marker as well as that of another retrotransposon *Noble225*, located in position 15.85–15.86 Mb between loci VVNTm5 and VVIu20.1.

The genotype of the certified blue-black-skinned clone PN162 was defined as genotype I. It was homozygous for 2 microsatellite markers and heterozygous for 11 as well as for the insertion of the two retrotransposons *Gret1* and *Noble225* (S3 Table). Only the 13 heterozygous loci were further considered. In the collection, two other blue-black and four grey-skinned Pinot clone also displayed genotype I. Conversely; the 26 remaining clones were polymorphic at one to nine of the 13 loci, leading to the identification of seven variant genotypes (genotypes II to VIII) (Table 1). Three blue-black-skinned clones shared genotype II showing allele 198 instead of allele 216 at VMC5g7 and three grey-skinned clones genotype III showing both alleles 198 and 216 at VMC5g7. All green-yellow-skinned clones displayed one of the five genotypes IV-VIII which polymorphism mainly result from the lack of one allele at loci from P2-106 to VMC7g3, among them, 12 clones shared genotype IV. Genotypes III and VIII displayed the same tri-allele profile at VMC5g7.

**Table 1:** Genotypes of the clones in studied at the 13 heterozygous loci.

Berry color	Clone nb	Chr. 2 genotypes	Locus														
			VMC5g7	SC8_0146_010	P2-106	P2-298	P2-442	VVNTm1	VVNTm2	*Gret1* insertion		VVNTm3	VVNTm5	*Noble225* insertion		VVIu20.1	VMC7g3
										Empty site	Full site			Empty site	Full site		
blue-black	3	I	188–216	123–128	222–262	118–129	110–126	161–168	378–387	1	1	272–296	288–300	1	1	363–386	116–132
blue-black	3	II	188–198	123–128	222–262	118–129	110–126	161–168	378–387	1	1	272–296	288–300	1	1	363–386	116–132
grey	4	I	188–216	123–128	222–262	118–129	110–126	161–168	378–387	1	1	272–296	288–300	1	1	363–386	116–132
grey	3	III	188–198–**216**	123–128	222–262	118–129	110–126	161–168	378–387	1	1	272–296	288–300	1	1	363–386	116–132
green-yellow	12	IV	188–198	123–128	222–262	118–129	110–126	**168**	**387**	**0**	**1**	272–296	288–300	1	1	363–386	116–132
green-yellow	3	V	188–216	123–128	222–262	118–129	**126**	**168**	**387**	**0**	**1**	**272**	**288**	**0**	**1**	**363**	**116**
green-yellow	3	VI	188–216	123–128	**262**	**129**	**126**	**168**	**387**	**0**	**1**	**272**	**288**	1	1	363–386	116–132
green-yellow	1	VII	188–198	123–128	222–262	118–129	110–126	161–168	378–387	0	1	272–296	288–300	1	1	363–386	116–132
green-yellow	1	VIII	188–198–**216**	123–128	222–262	118–129	110–126	161–168	378–387	0	1	272–296	288–300	1	1	363–386	116–132

Polymorphism is indicated in bold font.

### Haplotypes resolution of the L2 cell layer of the clones along chromosome 2

**Fig 1:**
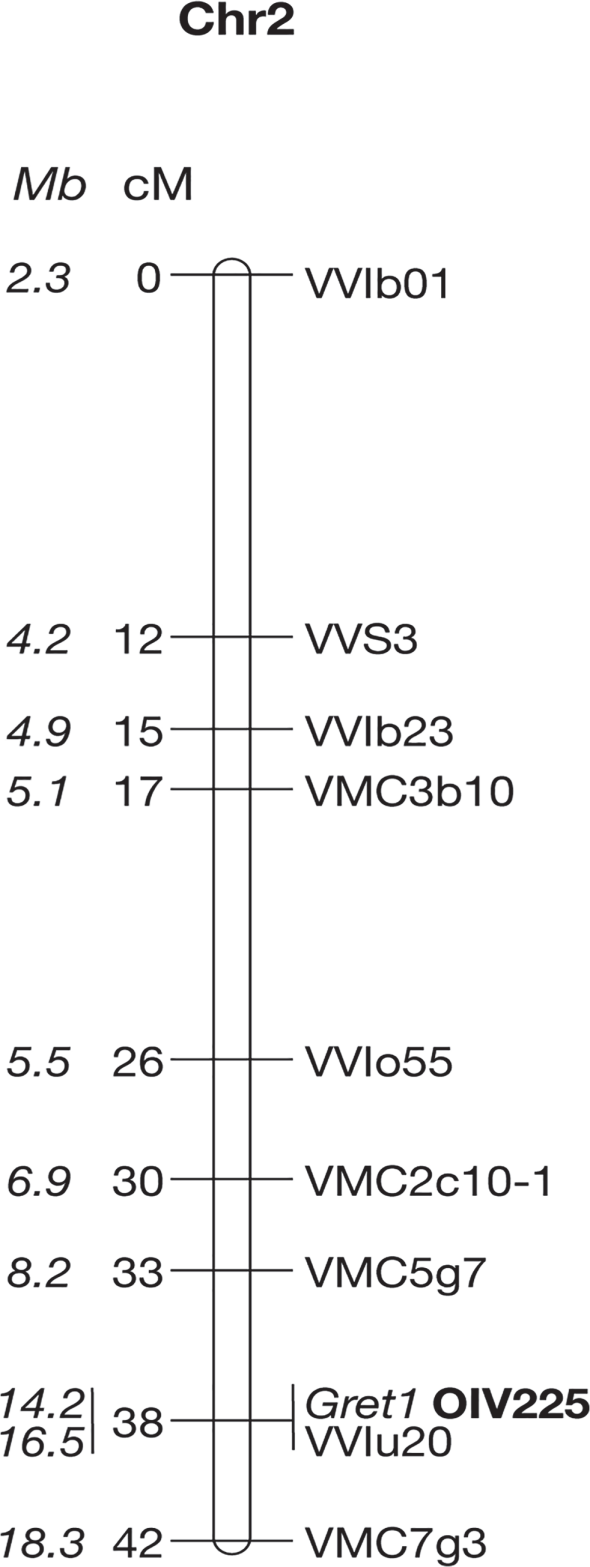
Genetic linkage map of the Pinot noir 162 chromosome 2. Positions of the markers along the chromosome 2 are given in cM and *Mb* (in italics).

Haplotypes were resolved by determining which alleles are linked together by genotyping self-progenies of the clones at the 13 heterozygous loci located from VMC5g7 to VMC7g3. The two respective haplotypes of genotype I were determined in a self-progeny of PN162. Fifty-one individuals among the fertile S1 offspring were both phenotyped for berry color and genotyped for the *Gret1* insertion. Thirty-five S1 offspring (68.6%) that produced blue-black berried clusters (OIV 225: 6) amplified the empty site of the *Gret1* insertion. Among them, eight that amplified only the empty site of *Gret1* were considered as homozygous while 27 that amplified both the empty and the full sites of *Gret1* were considered as heterozygous. Conversely, the remaining 16 S1 offspring (31,4%) that produced green-yellow berries (OIV 225: 1) that only amplified the full site of *Gret1* were considered as homozygous for the insertion. The genetic linkage map shows that the berry-color trait is a single locus co-locating with the *Gret1* insertion on chromosome 2, the green-yellow phenotype being recessive and associated with homozygous *Gret1* insertion (Fig. 1).

Ten non-recombinants individuals from loci VMC5g7 to VMC7g3 were subsequently genotyped at all heterozygous loci between the flanking markers. In addition, the polymorphic region of *VvMybA2* was sequenced. Alleles linked together with the *Gret1* insertion defined the canonical ‘white’ haplotype, referred as w216, according to the allele sized 216 bp at locus VMC5g7. Conversely, alleles linked together with the empty site of *Gret1* defined the canonical ‘colored’ haplotype referred as c188, *VvMybA1* being potentially functional. The 2-bp deletion (CA) in *VvMybA2* was linked together with the *Gret1* insertion while the lack of deletion was linked to the empty site of *Gret1*.

**Fig 2:**
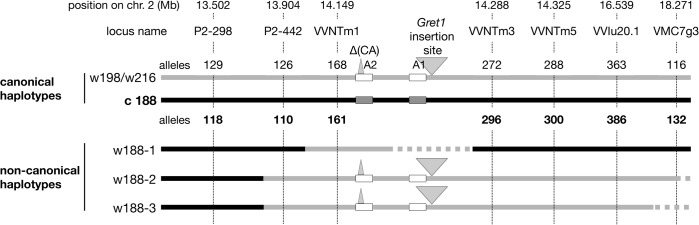
Schematic presentation of the Pinot haplotypes determined by genetic analysis. Solid grey line corresponds to the ‘white’ haplotype and solid black line to the ‘colored’ haplotype. Dotted lines symbolize deletion or unknown sequences. The boxes represent *VvmybA* genes: A1: *VvmybA1* and A2: *VvmybA2*. The grey triangle indicates the insertion. Positions on chromosome 2 are given en Mb according to the 12X genome sequence.

In the same way, haplotypes of eight clones were determined in their self-progenies, comprising 18 or 24 individuals. The selected clones were the following: three grey-skinned clones, PG52 and PG53 (genotype I) and PG3106 (genotype III) and five green-yellow-skinned clones, PB54 and PB55 (genotype IV), PB3009 and PB3172 (genotype V) and PB3232 (genotype VIII). As only the genetic information of the L2 cell layer is transmitted through sexual reproduction, only the haplotypes of the L2 cell layer could be determined. In the self-progeny of PG52, both alleles of loci from VMC5g7 to P2-442 displayed the expected Mendelian inheritance as well as those beyond VVNTm3, unlike alleles 168 at VVNTm1, 387 at VVNTm2, and *VvMybA2* displaying the 2-bp deletion (CA) that were shown by all siblings. Conversely, amplification of the full site of *Gret1* segregated and siblings that did not amplified the *Gret1* insertion specifically displayed alleles belonging to haplotype c188 upstream from P2-442 and downstream from VVNTm3. In addition, the empty site of *Gret1* was never amplified in these siblings. From this data, it was deduced that PG52 associated the canonical ‘white’ haplotype w216 and a new ‘white’ haplotype, w188-1, that displayed all alleles of canonical ‘colored’ haplotype c188, except alleles 168 at VVNTm1, 387 at VVNTm2 and *VvMybA2* displaying the 2-bp deletion (CA) that were those of the ‘white’ haplotype w216 and lacked the *Gret1* insertion. The four progenies of PG3106, PB54, PB55 and PB3232 showed the same Mendelian inheritance as PG52, except for VMC5g7 that segregated alleles 188 and 198, thus associating another ‘white’ haplotype w198 and the non-canonical ‘white’ haplotype, w188-1. Although the ‘white’ haplotypes w216 and w198 had different alleles at locus VMC5g7, both were considered as canonical ‘white’ haplotypes because they were identical at all other loci from SC8_0146_01 to VMC7g3. Conversely, in self-progenies of PB3009 and PB3172 all siblings displayed alleles belonging to w216 at all loci from P2-442 to VMC7g3 without segregation, including the *Gret1* full site, while they displayed a Mendelian inheritance for loci upstream from P2-298. The self-progeny of PG53 differed from the previous one by the segregation of allele 116 at VMC7g3 that was not amplified in 22% of the progeny. From this data, two additional non-canonical ‘white’ haplotypes were deduced, w188-2 and w188-3, that displayed alleles belonging to c188 from VMC5g7 to P-298 and alleles belonging to w216 (or w198) from P2-442 to VMC7g3, except haplotype w188-3 that had a null allele at VMC7g3 (Fig. 2).

The three non-canonical ‘white’ haplotypes w188-1, w188-2 and w188-3 differed more or less drastically from the canonical ‘white’ haplotypes by displaying part of the canonical ‘colored’ haplotype c188. These results can be explained by assuming that they have derived from the ‘colored’ haplotype c188 by the replacement of various sections of the ‘colored’ haplotype by the homologous section of the ‘white’ haplotype. In haplotype w188-1, the replaced region is located downstream from P2-442 and included allele 168 at VVNTm1, 387 at VVNTm2, and the *VvMybA2* polymorphic site thus a region longer than 30.89 kb. Moreover, as amplification of the *Gret1* empty site was impossible, the region of the haplotype w188-1 located between the *VvMybA2* polymorphic site and VVNTm3 that covered a maximum length of 107.85 kb was probably deleted.

The replaced section of the ‘colored’ haplotype by the ‘white’ haplotype in haplotype w188-2 spanned a region that extended from upstream P2-442 to downstream VMC7g3 probably to the end of the chromosome, corresponding to a region, which sized at least 4.367 Mb-long. Finally, in haplotype w188-3, the replacement of the ‘colored’ haplotype by the homologous ‘white’ haplotype extended from upstream P2-442 to downstream VVIu20.1 spanning a region of least 2.635 Mb-long. As locus VMC7g3 was not amplified, the region downstream VVIu20.1 could have been either deleted or replaced by an unknown sequence.

### Deduction of the genotypes of the L1 cell layer of the clones

After having resolved the structure of both haplotypes of the L2 cell layer, the haplotypes of the L1 cell layer were deduced to fit the different clone genotypes. Assuming that L1 cell layer of PN162 is identical to the L2, it results that genotype 1 associates haplotypes c188 and w216 in both cell layers (Table 2). The genotypes of the three Pinot gris clones combines a canonical and a non-canonical ‘white’ haplotypes in their L2 cell layer. Clone PG52 combines w216 with w188-1, PG3106 w198 with w188-1 and PG53 w216 with w188-3. This last association led to the partial homozygosity of the distal part of chromosome 2 from marker P2-442 to VVIu20.1, which includes the color locus. This result is consistent with the phenotype of the fertile self-progenies of the clones made up of 37 PG52 S1 and 61 PG53 S1 individuals all of them producing green-yellow-skinned berries (OIV 225: 1) [19]. Pinot gris being a chimera, L1 genotype is different from L2 genotype. Thus, PG52 can combine c188 with w216 in L1 and w188-1 and w216 in L2, PG53, c188 with w216 in L1 and w188-3 and w216 in L2. To confirm these hypotheses, 14 independent somaclones of PG52 and four of PG53 were regenerated. To identify from which cell layer the somaclones were regenerated, loci VVS2 and VVMD32 were analyzed in leaves and roots, an L2-derived tissue, of PG52, in its self-progeny and regenerated somaclones. These two loci were chosen because they showed a tri-allele combination in PG52 leaves, 126-134-148 and 236-252-268, at VVS2 and VVMD32 respectively. The PG52 roots exhibited the di-allele combinations 126-134 and 236-252, respectively; the same alleles segregated in the S1 progeny [19]. On the contrary, PG52 somaclones leaves displayed 134-148 and 236-268, respectively. These allelic combinations differing from those of the roots and S1 progeny suggested a L1 origin of PG52 somaclones. To confirm this origin, PG52 and PG53 somaclones were brought to fructification and shown to produce blue-black-skinned berries (OIV 225: 6) that were different from the parental grey berries (OIV 225: 4) and from the S1 progenies (OIV 225: 1) [19]. Both genotype and phenotype analyses suggested that somaclones were originated from the L1 cell layer. Finally, PG3106 can combine haplotypes c188 and w216 in L1 and haplotypes w188-1 and w198 in L2, leading to the tri-allele profile observed at locus VMC5g7 (188-196-216).

**Table 2:** Association of haplotypes in the L1 and L2 cell layers to reconstitute the genotypes of nine of the clones in study.

Clones ID	Chr. 2 genotype	Cell layer	Haplotypes	
**PN162**	I	L2	w216	c188
		expected L1	w216	c188
**PG52**	I	L2	w216	w188-1
		expected L1	w216	c188
**PG53**	I	L2	w216	w188-3
		expected L1	w216	c188
**PG3106**	III	L2	w198	w188-1
		expected L1	w216	c188
**PB54**	IV	L2	w198	w188-1
		expected L1	w198	w188-1
**PB55**	IV	L2	w198	w188-1
		expected L1	w198	w188-1
**PB3009**	V	L2	w216	w188-2
		expected L1	w216	w188-2
**PB3172**	V	L2	w216	w188-2
		expected L1	w216	w188-2
**PB3232**	VIII	L2	w198	w188-1
		expected L1	w216	unknown

Similarly, genotypes of both cell layers of the five Pinot blanc clones were deduced. To explain genotype IV, PB54 and PB55 that associated haplotypes w198 and w188-1 in their L2 cell layer were likely to have the same haplotype combination in the L1 cell layer. Similarly, PB3009 and PB3172 that displayed genotype V can combine w216 and w188-2 in both cell layers. Finally, PB3232, which displayed genotype VIII and combined w198 with w188-1 in the L2 cell layer is likely to have another haplotype combination of in its L1 cell layer to explain the tri-allele profile at locus VMC5g7 (188-196-216) and the di-allele profiles at all other loci downstream from VMC5g7. It probably combined w216 with an unknown ‘white’ haplotype displaying the alleles of c188 at all locus downstream from VVNTm1. In addition, a deletion can be responsible for the lack of amplification of the *VvMybA* genes at the color locus as well as of the empty site of *Gret1*. Thus, PB3232 is most likely a chimera.

### Analysis of two Pinot gris clone and their spontaneous bud sports

The genotypes of two Pinot gris clone PGMA19 and BCPG9.S7.1 and of their respective bud sports were determined: the green-yellow PGMA19.S5 and BCPG9.S7.2 and the blue-black PGMA19.S6 bud sports. The two grey clones as well as the blue-black bud sport displayed genotype I while the green-yellow bud sports genotype VI, showing one allele from locus P2-106 to VVNTm5, while loci upstream from P2-106 and downstream VVNTm5 including the *Noble225* insertion site were heterozygous. Genotypes of the stem pith of the Pinot gris, a L2-derived tissue, also displayed genotype VI.

Genotype VI can be explained by the association of the canonical haplotype w216 with a new non-canonical haplotype stemmed from the replacement of a section of the ‘colored’ haplotype along a region spanning markers P2-106 to VVMTM5 including the color locus, thus at least 1,357 Mb in length, by the homologous section of the ‘white’ canonical haplotype. However, a hemizygous situation resulting from a large deletion cannot be excluded. As the genotype of the green-yellow bud sports is identical to that of the stem pith of the two Pinot gris clones, it confirmed the origin of the green-yellow bud sports from Pinot gris by invasion of epidermal colored cells by subepidermal white cells.

## Discussion

The molecular origin of clonal variation in grapevine remains a long-standing question. Focusing on the distal extremity of chromosome 2 including the berry-color locus, we have identified polymorphic genotypes in a collection of Pinot clones. By resolving their respective haplotypes, we have shown that at least six haplotypes could account for the loss of anthocyanin biosynthesis, four of them resulting from chromosome replacement and deletion events.

In a previous study carried out on a collection of Pinot clones to understand the structural dynamics along chromosome 2, Vezzulli et al. (2012) have observed an homozygous-like region in the genotypes of the berry flesh and roots of one Pinot gris and of two clones of Pinot blanc. This result was consistent with the presence of deletions, extending for at least 4.2 Mb for L2 cell layer Pinot gris while ranging from 100–179 kb for both cell layers of Pinot blanc. Finally, they made the assumption that Pinot blanc and Pinot gris arose independently from the ancestral Pinot noir, suggesting a parallel evolutionary model [20].

While we observed similar genotypes in our collection of Pinot clones, we have tackled the issue of the genetic origin of these genotypes by resolving the structure of the haplotypes of nine clones by an allele segregation analysis in clone self-progenies. As gametes are formed from the L2 cell layer, only haplotypes of this cell layer could be determined. First, both haplotypes of the clone of Pinot noir 162, chosen as reference, were determined by identifying which alleles at 13 loci were linked together. It combined the canonical ‘white’ haplotype w216, showing the expected *Gret1* insertion upstream from *VvmybA1* and the 2-bp deletion (CA) in the coding sequence of *VvmybA2*, and the canonical ‘colored’ haplotype c188, devoid of both the *Gret1* insertion and CA deletion, allowing anthocyanin biosynthesis. The same experiment was then carried out for five Pinot blanc and three Pinot gris and their haplotypes were compared to those of the Pinot noir 162. In particular, this approach was used to clarify whether genotypes displaying only one allele at a given locus were homozygous or hemizygous, referring to the presence of null alleles. We identified another canonical ‘white’ haplotypes showing the expected insertion of *Gret1* retrotransposon, but displaying allele 198 instead of 216 at locus VMC5g7. In addition, three non-canonical ‘white’ haplotypes were shown to stem from the replacement of different sections of the canonical ‘colored’ haplotype by its ‘white’ homolog that had in common allele 168 at VVNTm1 and allele 387 at VVNTm2.

Such rearrangements lead to chimeric haplotypes comprising sections of the ‘white’ haplotype and sections of the ‘colored’ haplotype. In colorless L2 cell layer of Pinot gris and Pinot blanc clones, different associations involving a canonical and a non-canonical ‘white’ haplotypes were observed leading to partial homozygosity, along a region sized from 31 kb to 4,4 Mb of chromosome 2, thus in a loss of heterozygosity. Homozygous and hemizygous situations together were also observed. The association of a canonical white haplotype with haplotype w188-1 observed in the L2 cell layer genotype of three clones of Pinot blanc (PB54, PB55 and PB3232) as well as of two clones of Pinot gris (PG52 and PG3106) leads to genotypes homozygous on a region 31 kb-long that include alleles 168 at VVNTm1, 387 at VVNTm2, and the mutated allele of *VvMybA2* followed by a 108 kb-long hemizygous region, probably corresponding to a deletion including the functional *VvMybA1* gene. It is possible that a same type of haplotype showing a 260 kb-long deletion in the colored allele of Cabernet Sauvignon, a blue-black-skinned variety, is responsible for the phenotype of Shalistin and of the L2 cell layer of Malian, two bud sports of Cabernet Sauvignon, which are green-yellow and bronze-berried, respectively [17]. Meanwhile, the association of the canonical w216 haplotype with haplotype w188-2, observed in the L2 cell layer genotype of the two Pinot blanc clones, PB3009 and PB3172, leads to a genotype homozygous on the distal section of chromosome 2 along a region spanning at least 4.367 Mb. Finally, the association of the canonical w216 haplotype with haplotype w188-3 observed in the L2 cell layer genotype of the Pinot gris clone PG53 leads to a genotype homozygous on a region longer than 2.24 Mb, followed by an hemizygous state of the distal part of chromosome 2 including marker VMC7g3 that probably results from a deletion.

In addition, genotypes of two Pinot gris clones (PGMA19 and BCPG9.S7.1) and of their respective green-yellow-berried bud sports could be ascribed to the replacement of a section of the ‘colored’ haplotype along a 176 kb-long region, including the color locus, by the homologous section of ‘white’ haplotype. Nevertheless, the hypothesis of a large deletion cannot be excluded to explain the white phenotype of the L2 of the Pinot gris clone and of their green-yellow bud-sports.

### How were the non-canonical haplotypes formed?

**Fig 3:**
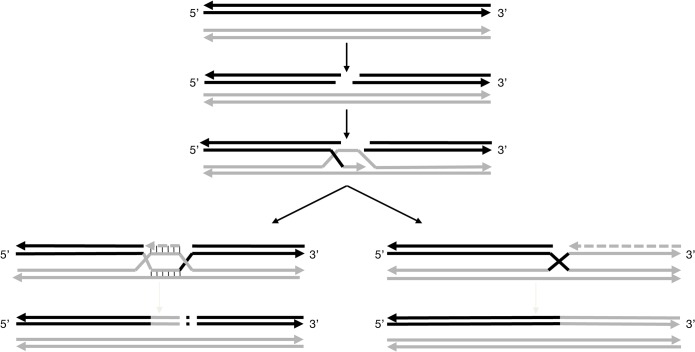
Models for pathways proposed to explain the non-canonical ‘white’ haplotypes. These models are based on the repair of DSBs. After induction of the double-strand break in the acceptor molecule, in that case the ‘colored’ haplotype (solid black line), the ends are processing to yield 3’single-strands tails. Then, the 3’ends invades the double-stranded donor molecule, here the ‘white’ haplotype (solid grey line) and repair synthesis occurs. For the further processing of the intermediate two possible outcomes can be envisaged. For the formation of w188-1: the acceptor molecule is elongating, possibly up to the homology of the second 3’end of the DSB followed by the insertion of a genomic sequence copied from elsewhere into the break. For the formation of w188-2: the acceptor molecule is elongating up to the end of the chromosome using the invading donor sequence as template.

The non-canonical ‘white’ haplotypes consisting in the replacement of a more or less extended section of the ‘colored’ haplotype by the ‘white’ haplotype could have been generated by gene conversion which represents the non-reciprocal transfer of information between two homologous sequences to duplicate one of the haplotype, with the corresponding loss of the other [35]. Gene conversion operates during replicative DNA synthesis and is well documented in yeast [36], rice [37] as well as in human [38]. The model of recombination starts by a double-strand break (DSB) in the recipient molecule, in our case the ‘colored’ haplotype (Fig. 3). Then, one end of the DSB invades the homologous chromosome, the ‘white’ haplotype, and repairs the break using the sequence of the homolog as template. The invading strand can reach the end of the chromosome replacing the recipient molecule by the donor sequence. Such a mechanism can explain haplotype w188-2. If the process stops before the end of the chromosome, it results in a loss of information on the recipient haplotype as in the case of haplotype w188-3, truncated beyond marker VVIu20.1. The extension of the molecule can also stop before the end of the chromosome where the recipient sequence is recovered. Such a mechanism can explain the probable non-canonical ‘white’ haplotype giving the particular genotypes of the white bud sports and of the L2 cell layer of their grey parents.

Haplotype w188-1 can result from another mechanism also beginning by a DSB in the ‘colored’ haplotype followed by template copying of the homolog chromosome; however, the process ends shortly, possibly at a second site of DSB, where the chromosome is rejoined in a manner that deletes a portion of the chromosome. Classically, this type of repair is associated with deletions, but also insertions due to copying sequences from elsewhere into the break [32]. In the case of w188-1, the deleted region includes the *VvMybA1* gene.

It is possible that the ‘colored’ haplotype of chromosome 2 is prone to initiate DSB along an extended region between markers SC8-0146-10 (12.67 Mb) and VVNTm1 (14.15 Mb), a region probably different from the centromere that can be localized around position 11.5 Mb according to a high density of repeated sequences and transposable elements and a low level of coding sequences [13]. This could explain why the breaking points were different in haplotypes w188-1 and w188-2 or w188-3 and in the genotype of the L2 cell layer of PGMA19 and BCPG9.S7.1 and their green-yellow bud-sports.

Previous results tend to show that the ‘white’ haplotype have a selective advantage over the ‘colored’ one. Indeed, almost all black-berried varieties are heterozygous at the *VvMybA* locus. Only nine *V*. *vinifera* varieties upon 137 appeared homozygous for the ‘colored’, as a consequence from the successful spread of the ‘white’ haplotype among varieties by crossing after domestication [8]. The possible selective advantage of the ‘white’ haplotype could result from the deleterious effect of anthocyanin pigment at high concentration and/or from the huge metabolic cost of its synthesis [39].

### How are somatic mutations fixed in chimeric clones?

**Fig 4:**
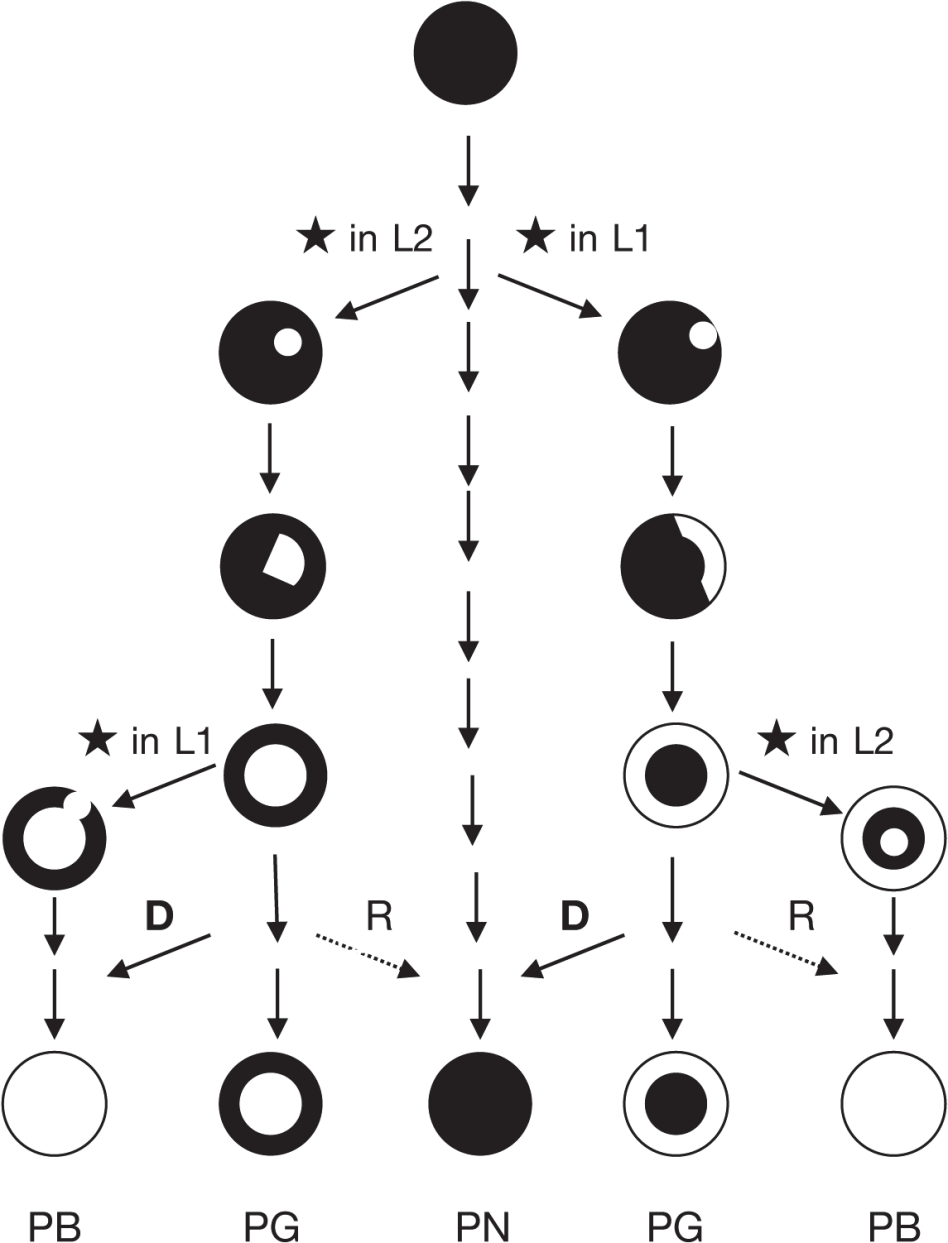
Scheme of evolutionary mechanism of the pinot clones. The propagation of a mutated cell of the meristem (★ causes the formation of a mericlinal sector, then the complete invasion of a cell layer created a periclinal chimera. Cellular displacement from the L2 to the L1 cell layers (D) and less frequent cellular rearrangements (R) from the L1 to the L2 cell layers lead to the homogenization of the cell layers and to the loss of the chimeric state. PB: Pinot blanc, PG: Pinot gris, PN Pinot noir.

To be retained, the double-strand break must first have occurred in a meristemic cell undergoing active mitosis because if it takes place in a non-meristematic cell unable of cellular differentiation the mutation is ultimately lost. The event can occur randomly in the L1 or in the L2 cell layer. After DSBs are repaired, the mutant cell can survive and eventually proliferate according to its cell layer, giving rise in a first step to a mericlinal chimera displaying a mutated sector. When a new meristem is formed from this mutated sector, the mutated cell layer becomes homogenous in the entire cell layer of the newly formed stem that is a rather stable periclinal chimera in which the mutation is fixed (Fig. 4).

The phenotypic instability of chimeric plants can account for cellular rearrangements in the chimera. An invasion by cells from the inner layers into the outer L1 layer, termed “displacement”, can occur owing to the low level of organization of cell division in the inner layers [40]. Displacement may have occurred to generate the green-yellow-shinned bud sports from Pinot gris via the invasion of epidermal colored cells (L1) by subepidermal white cells (L2) mutated at the berry-color locus. Moreover, Pinot blanc clones PB54 and PB55, which share with Pinot gris clone PG3106 the same L2 cell layer genotype could also result from a displacement that took place in the Pinot gris. None of the three clones of Pinot gris which L2 cell layer haplotypes were determined presented haplotype w188-2; however, Pinot gris clones SMA505 and SMA514 described by Vezzuli et al. can display this haplotype in their L2 cell layer [20]. Displacement can also explain the appearance of Shalistin from Malian [6]. The opposite phenomenon, L1 cell invasion of the inner layer, a process called “replacement”, is accepted as being rare in angiosperms, owing to the stability of the anticlinal cell divisions. However, the blue-black-skinned bud sport of PGMA19 could result from a replacement event. In both cases, the cell rearrangements have led to the homogenization of the cell layers and the loss of the chimeric state (Fig. 4).

### Are all Pinots blanc the result of cell rearrangements of Pinot gris?

Chimerism of Pinot gris clone is well documented [19,20]; however, tri-allele profiles at locus VMC3c9, VrZAG25, VVS2 and VMC5g7 provide evidence that Pinot blanc can also be chimeric. In particular, PB3232 that shows genotype VIII is characterized by the tri-allele profile at VMC5g7 (188-198-216) and the di-allele profiles at loci VVNTm1 (161–168) and VVNTm2 (378–387). As its L2 cell layer combines haplotypes w198 and w188-1, the L1 cell layer of PB3232 is likely to consist in the combination of the canonical haplotype w216 associated with an uncharacterized ‘white’ haplotype displaying allele 161 at VVNTm1 and 378 at VVNTm2, both alleles being specific of the ‘colored’ haplotype. Instead of cellular rearrangements, this situation may have arisen from independent mutations of the ‘colored’ locus in the L1 and the L2 cell layers of this Pinot gris clone. A combination of w198 with the same uncharacterized ‘white’ haplotype could also explain genotype VII diallelic at all locus along the distal arm of chromosome 2. A structure consisting of a white L1 cell layer and a colored L2 cell layer has never been demonstrated. Nevertheless, such a structure can exist without having been identified, the grapes of such a structure being identical to those of Pinot noir.

Pinot noir is one of the most ancient varieties multiplied by vegetative propagation during at least six centuries since the first mentions of Pinot by name, and maybe since the Roman times according to Columella’s description. It is accepted that the original seedling was Pinot noir and it is most likely that the genotype of the modern Pinot noir is close to that of the ancestor except for all spontaneous mutations that have been able to accumulate since. These mutations have contributed to the great diversity of the clones valuable today to adapt the culture of Pinot noir to many vineyards and to produce a wide range of wines, all renowned. As we showed, different clones of Pinot gris and from them Pinot blanc appeared independently by major events of chromosome replacements and deletions resulting from gene conversion. These events confirm the proclivity of pinot toward spontaneous mutations, in particular the proclivity of an extended region the ‘colored’ haplotype to initiate DSB. Moreover, as these events result in a targeted selective sweep eliminating the haplotype carrying the functional genes of the color berry, it may enhance the possible selective advantage of the ‘white’ haplotype. Thus, these events are the driving forces behind the genetic drift of clones and the evolution of the grapevine genome.

## Supporting Information

S1 Table List of the introductions maintained in germplasm repositories at INRA-Colmar. Certified clones are indicated by *.
(DOCX)

S2 Table The most prevalent genotype in the Pinot collection. Linkage groups according to the reference map of Doligez et al. (2006).
(DOCX)

S3 Table Loci on the distal region of chromosome 2. Their respective position according to the 12X genome sequence and genotype of PN162 are given. * two bands are amplified from the forward priming site because of a duplication in the *VvMybA2* gene.
(DOCX)

S4 Table Primer sequences of five markers mapped in the distal region of chromosome 2. (DOCX)

S5 Table Genotypes of the 33 clones in study at 7 polymorphic loci independent from the color locus. Polymorphism is indicated in bold.
(DOCX)
