# Supplementary material for: Chromosome Replacement and Deletion Lead to Clonal Polymorphism of Berry Color in Grapevine
Source: PLoS Genet. 2015 Apr 2;11(4):e1005081. doi: 10.1371/journal.pgen.1005081 (PMC4383506; doi:10.1371/journal.pgen.1005081)
Supplement: S1 Table — Certified clones are indicated by *. (DOCX) [file pgen.1005081.s001.docx]

| Clone | Berry color | Chr.2 genotype | Repository ID | Origin |
| --- | --- | --- | --- | --- |
| PN162* | Blue-black | I | CTPS162 | Alsace |
| PN292* | Blue-black | I | CTPS292 | Jura |
| PN871* | Blue-black | II | CTPS871 | Champagne |
| PN3023 | Blue-black | II | 193.Col.3023 | Alsace |
| PN3042 | Blue-black | II | 193.Col.3042 | Alsace |
| PGMA19.S6 | Blue-black | I | 195.Col.BPGMA19.S6 | Burgundy |
| PG52* | Grey | I | CTPS 52 | Alsace |
| PG53* | Grey | I | CTPS 53 | Alsace |
| PG3028 | Grey | III | 195.Col.3028 | Alsace |
| PG3106 | Grey | III | 195.Col.3106 | Alsace |
| PG3112 | Grey | III | 195.Col.3112 | Alsace |
| BCPG9.S7.1 | Grey | I | 195.Col.BCPG9 | Burgundy |
| PGMA19 | Grey | I | 195.Col.BPGMA19 | Burgundy |
| PB54* | Green-yellow | IV | CTPS 54 | Alsace |
| PB55* | Green-yellow | IV | CTPS 55 | Alsace |
| PB3003 | Green-yellow | IV | 194.Col.3003 | Alsace |
| PB3009 | Green-yellow | V | 194.Col.3009 | Alsace |
| PB3020 | Green-yellow | IV | 194.Col.3020 | Alsace |
| PB3068 | Green-yellow | IV | 194.Col.3068 | Alsace |
| PB3150 | Green-yellow | IV | 194.Col.3150 | Alsace |
| PB3163 | Green-yellow | VII | 194.Col.3163 | Alsace |
| PB3172 | Green-yellow | V | 194.Col.3172 | Alsace |
| PB3183 | Green-yellow | IV | 194.Col.3183 | Alsace |
| PB3186 | Green-yellow | V | 194.Col.3186 | Alsace |
| PB3188 | Green-yellow | IV | 194.Col.3188 | Alsace |
| PB3189 | Green-yellow | IV | 194.Col.3189 | Alsace |
| PB3191 | Green-yellow | IV | 194.Col.3191 | Alsace |
| PB3209 | Green-yellow | IV | 194.Col.3209 | Alsace |
| PB3226 | Green-yellow | IV | 194.Col.3226 | Alsace |
| PB3232 | Green-yellow | VIII | 194.Col.3232 | Alsace |
| PB013 | Green-yellow | VI | 194.Col.3 | Burgundy |
| BCPG9.S7.2 | Green-yellow | VI | 195.Col.BCPG9.S7.2 | Burgundy |
| PGMA19.S5 | Green-yellow | VI | 195.Col.BPGMA19.S5 | Burgundy |
