# Supplementary material for: Chromosome Replacement and Deletion Lead to Clonal Polymorphism of Berry Color in Grapevine
Source: PLoS Genet. 2015 Apr 2;11(4):e1005081. doi: 10.1371/journal.pgen.1005081 (PMC4383506; doi:10.1371/journal.pgen.1005081)
Supplement: S2 Table — Linkage groups according to the reference map of Doligez et al. (2006). (DOCX) [file pgen.1005081.s002.docx]

| linkage group | locus | genotype |
| --- | --- | --- |
| 1 | VMC4d9-2 | 235-237 |
|  | VMC7g5 | 182 |
|  | VMC8a7 | 157 |
|  | VVS29 | 167-176 |
| 2 | VMC3b10 | 112-123 |
|  | VMC2c10 | 148-170 |
|  | VMC6b11 | 79-89 |
|  | VrZAG93 | 184 |
|  | VVS3 | 214 |
| 3 | VMC8f10 | 196-198 |
|  | VVMD28 | 218-236 |
|  | VVMD36 | 251 |
| 4 | VMC2e10 | 52-67 |
|  | VVMD32 | 236-268 |
| 5 | VrZAG79 | 234-241 |
|  | VVMD27 | 183-187 |
| 6 | VMC2h9 | 116-118 |
|  | VrZAG30 | 145-147 |
|  | VVS5 | 116-142 |
| 7 | VrZAG62 | 186-193 |
|  | VVMD31 | 212 |
|  | VVMD6 | 197 |
|  | VVMD7 | 236-240 |
| 8 | VMC2f12 | 192 |
|  | VMC3c9 | 256-263 |
|  | VVS4 | 165-170 |
| 9 | VMC3g8-2 | 164-175 |
|  | VMC4a5 | 205-214 |
| 10 | VrZAG25 | 225-237 |
|  | VrZAG67 | 125-151 |
| 11 | VMC2a12 | 101-118 |
|  | VVMD25 | 240-250 |
|  | VVMD8 | 134-136 |
|  | VVS2 | 134-148 |
| 12 | VMC2h4 | 200-234 |
|  | VMc8g6 | 148-170 |
| 13 | VMC3b12 | 106-108 |
|  | VMC3d12 | 198-214 |
|  | VVS1 | 179-187 |
| 14 | VMC2c3 | 164-193 |
|  | VMC6c10 | 110-127 |
|  | VVMD24 | 214-216 |
| 15 | VVMD30 | 102-124 |
| 16 | VMC1e11 | 192-204 |
|  | VVMD5 | 224-235 |
| 17 | VMC2h3 | 70 |
|  | VMC3a9 | 79 |
| 18 | VMC2a7 | 94-96 |
|  | VMC7f2 | 197 |
| 19 | VMC3b7-2 | 101-103 |
|  | VMC5h11 | 184-200 |
