# Supplementary material for: Chromosome Replacement and Deletion Lead to Clonal Polymorphism of Berry Color in Grapevine
Source: PLoS Genet. 2015 Apr 2;11(4):e1005081. doi: 10.1371/journal.pgen.1005081 (PMC4383506; doi:10.1371/journal.pgen.1005081)
Supplement: S3 Table — Their respective position according to the 12X genome sequence and genotype of PN162 are given. * two bands are amplified from the forward priming site because of a duplication in the VvMybA2 gene. (DOCX) [file pgen.1005081.s003.docx]

| Locus | Location on chr. 2 (Mb) | Genotype of PN162  (c-w) |
| --- | --- | --- |
| VMC5g7 | 8.223 | 188-216 |
| SC8_0146_010 | 12.674 | 128-123 |
| P2-106 | 12.968 | 222-262 |
| SC8_0146_026 | 12.970 | 254 |
| P2-298 | 13.502 | 118-129 |
| P2-442 | 13.904 | 110-126 |
| VVNTm1 | 14.149 | 161-168 |
| VVNTm2 | 14.151 | 378-387 |
| *VvMybA2* | 14.180 | 132/412-130/410* |
| *Gret1* insertion | 14.240 - 14.251 | empty site-full site |
| VVNTm3 | 14.288 | 296-272 |
| VVNTm4 | 14.296 | 206 |
| VVNTm5 | 14.325 | 300-288 |
| *Noble225* insertion | 15.854 - 15.859 | empty site-full site |
| VVIu20.1 | 16.539 | 386-363 |
| VMC7g3 | 18.271 | 132-116 |
