# Supplementary material for: Chromosome Replacement and Deletion Lead to Clonal Polymorphism of Berry Color in Grapevine
Source: PLoS Genet. 2015 Apr 2;11(4):e1005081. doi: 10.1371/journal.pgen.1005081 (PMC4383506; doi:10.1371/journal.pgen.1005081)
Supplement: S4 Table — (DOCX) [file pgen.1005081.s004.docx]

| Locus | | | sense | | Primer sequence | |
| --- | --- | --- | --- | --- | --- | --- |
| SSR P2-106 | | | | forward | | GCTTATTAGCATTGATTGGAAA |
|  |  |  |  | reverse | | CTTTGACACTTCATGCTTGGTA |
| SSR P2-298 | | | | forward | | GGATTTTCCATCCTTCTTTGC |
|  |  |  |  | reverse | | TGCACCAAGCATAAGCACTC |
| SSR P2-442 | | | | forward | | TTAGAGGACAATAAAACATG |
|  |  |  |  | reverse | | GAGGGAGATAATTCTTATTA |
| *VvMybA2* | |  | | forward | | AGCCTCACCCTCACAAGTTC |
|  | |  | | reverse | | TGTCATCATTCGGTTGTGGT |
| *Noble225 insertion* | 5' full site | | | forward | | AGAGTAGACTAAATGCTTGGAA |
|  |  |  |  | reverse | | TCCTAAATGATCTCTCCTTTT |
|  | 3' full site | | | forward | | TTTTCTTATTCAGTCATTTGTG |
|  |  |  |  | reverse | | TTCAAGGTGTTCTTCTTACA |
|  | empty site | | | forward | | AGAGTAGACTAAATGCTTGGAA |
|  |  |  |  | reverse | | TTCAAGGTGTTCTTCTTACA |
