# Supplementary material for: Chromosome Replacement and Deletion Lead to Clonal Polymorphism of Berry Color in Grapevine
Source: PLoS Genet. 2015 Apr 2;11(4):e1005081. doi: 10.1371/journal.pgen.1005081 (PMC4383506; doi:10.1371/journal.pgen.1005081)
Supplement: S5 Table — Polymorphism is indicated in bold. (DOCX) [file pgen.1005081.s005.docx]

| **Berry**  **color** | **clone ID** | **Locus** | | | | | | |
| --- | --- | --- | --- | --- | --- | --- | --- | --- |
|  |  | **VMC3b12** | **VMC3c9** | **VMC7g5** | **VrZAG25** | **VVMD7** | **VVMD8** | **VVS2** |
| Blue-black | PN292 | 106-108 | 256-263 | 182 | 225-237 | 236-240 | 134-136 | 134-148 |
|  | PN871 |  |  |  |  |  |  |  |
|  | PN3023 |  |  |  |  |  |  |  |
|  | PN3042 |  |  |  |  |  |  |  |
|  | PGMA19.S6 |  |  |  |  |  |  |  |
|  | PN162 |  |  |  |  |  | 134-136-**138** |  |
| Grey | PG53 | 106-108 | 256-263 | 182 | 225-237 | 236-240 | 134-136 | 134-148 |
|  | PGMA19 |  |  |  |  |  |  |  |
|  | BCPG9.S7.1 |  |  |  |  |  |  |  |
|  | PG52 |  |  |  |  |  |  | **126**-134-148 |
|  | PG3028 | 106-108-**116** |  |  |  | **230**-236-240 |  |  |
|  | PG3106 |  |  |  |  |  |  |  |
|  | PG3112 |  |  |  |  |  |  |  |
| Green-yellow | PB54 | 106-108 | 256-263 | 182 | 225-237 | 236-240 | 134-136 | 134-148 |
|  | PB55 |  |  |  |  |  |  |  |
|  | PB3003 |  |  |  |  |  |  |  |
|  | PB3009 |  |  |  |  |  |  |  |
|  | PB3020 |  |  |  |  |  |  |  |
|  | PB3163 |  |  |  |  |  |  |  |
|  | PB3172 |  |  |  |  |  |  |  |
|  | PB3183 |  |  |  |  |  |  |  |
|  | PB3186 |  |  |  |  |  |  |  |
|  | PB3188 |  |  |  |  |  |  |  |
|  | PB3232 |  |  |  |  |  |  |  |
|  | PB013 |  |  |  |  |  |  |  |
|  | PGMA19.S5 |  |  |  |  |  |  |  |
|  | BCPG9.S7.2 |  |  |  |  |  |  |  |
|  | PB3068 |  |  |  |  |  |  | 134-148-**150** |
|  | PB3150 |  | 256-263-**268** |  |  |  |  |  |
|  | PB3189 |  |  |  | 225-237-**245** |  |  |  |
|  | PB3191 |  |  |  |  |  |  |  |
|  | PB3209 |  |  |  |  |  |  |  |
|  | PB3226 |  |  | **168**-182 |  |  |  |  |
